# Supplementary material for: Saccharomyces boulardii (CNCM I-745) improves intestinal damage in sepsis by remodeling intestinal flora structure
Source: Front Cell Infect Microbiol. 2025 Jul 31;15:1602792. doi: 10.3389/fcimb.2025.1602792 (PMC12350329; doi:10.3389/fcimb.2025.1602792)
Supplement: Supplementary file 1 [file DataSheet1.zip › summary/summary/7_Differential_analysis/1_diff_abundance/4_Order/Group.DW4_vs_BW4/Group.DW4_vs_BW4_diff_barplot.pdf]

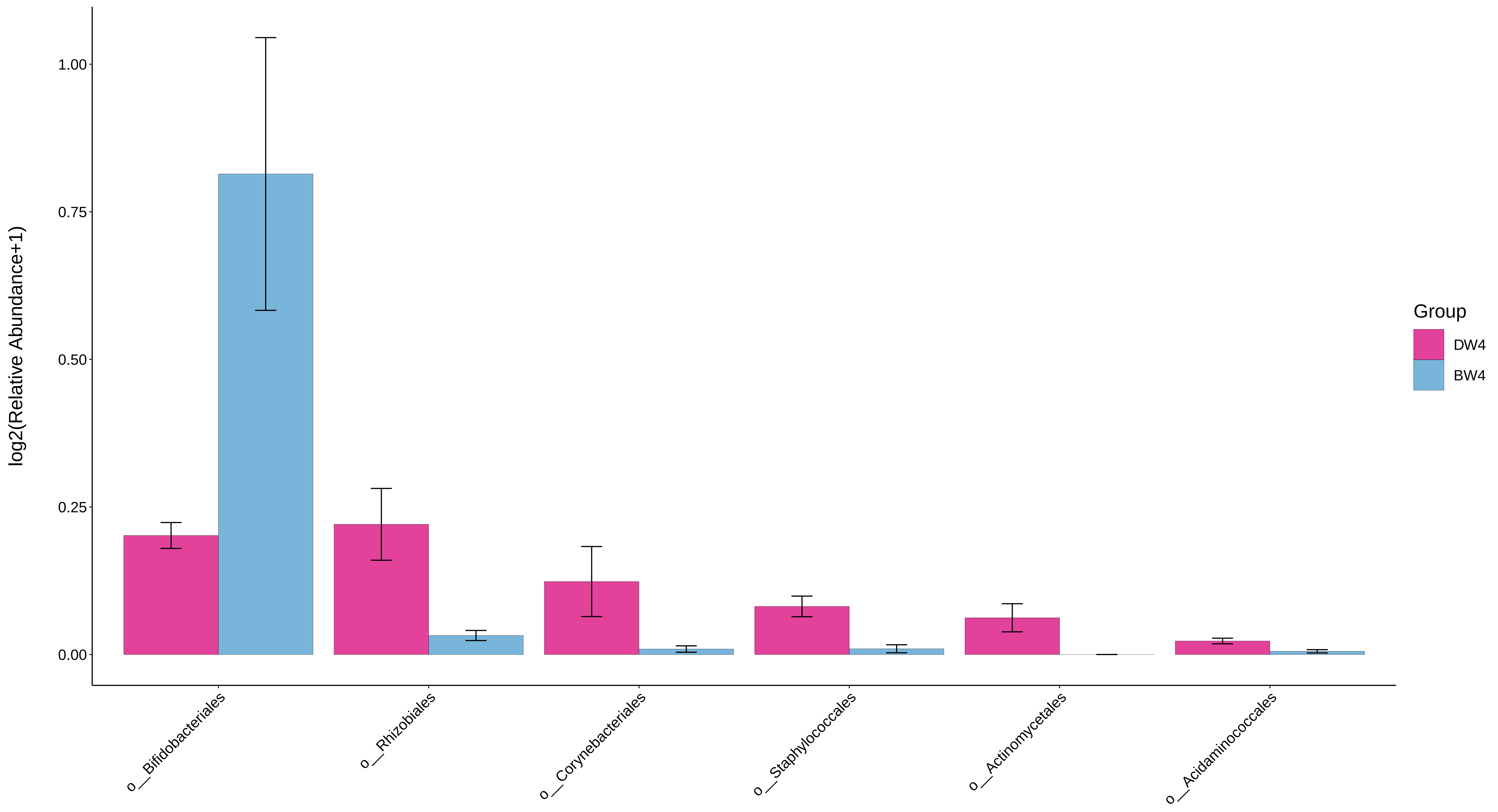
log2(Relative Abundance+1)
1.00
0.75
0.50
0.25
0.00
o__Bifidobacteriales
o__Rhizobiales
o__Corynebacteriales
o__Staphylococcales
o__Actinomycetales
o__Acidaminococcales
Group
DW4
BW4
